# Nanohollow Titanium Oxide Structures on Ti/FTO Glass Formed by Step-Bias Anodic Oxidation for Photoelectrochemical Enhancement

**DOI:** 10.3390/nano12111925

**Published:** 2022-06-04

**Authors:** Chi-Hsien Huang, Yu-Jen Lu, Yong-Chen Pan, Hui-Ling Liu, Jia-Yuan Chang, Jhao-Liang Sie, Dorota G. Pijanowska, Chia-Ming Yang

**Affiliations:** 1 Department of Materials Engineering, Ming Chi University of Technology, New Taipei 243, Taiwan; chhuang@mail.mcut.edu.tw (C.-H.H.); eharoger@gmail.com (J.-L.S.); 2 Department of Neurosurgery, Chang Gung Memorial Hospital at Linkou, Taoyuan City 333, Taiwan; alexlu0416@gmail.com; 3 The College of Medicine, Chang Gung University, Taoyuan City 333, Taiwan; 4 Department of Electronic Engineering, Chang Gung University, Taoyuan City 333, Taiwan; pan890808@gmail.com (Y.-C.P.); hll20121219@gmail.com (H.-L.L.); d0827110@cgu.edu.tw (J.-Y.C.); 5 Nalecz Institute of Biocybernetics and Biomedical Engineering, Polish Academy of Sciences, 02-109 Warsaw, Poland; 6 Institute of Electro-Optical Engineering, Chang Gung University, Taoyuan City 333, Taiwan; 7 Biosensor Group, Biomedical Engineering Research Center, Chang Gung University, Taoyuan City 333, Taiwan; 8 Artificial Intelligence Research Center, Chang Gung University, Taoyuan City 333, Taiwan

**Keywords:** TiO_x_, anodic oxidation, photoelectrochemical, nanohollows

## Abstract

In this study, a new anodic oxidation with a step-bias increment is proposed to evaluate oxidized titanium (Ti) nanostructures on transparent fluorine-doped tin oxide (FTO) on glass. The optimal Ti thickness was determined to be 130 nm. Compared to the use of a conventional constant bias of 25 V, a bias ranging from 5 V to 20 V with a step size of 5 V for 3 min per period can be used to prepare a titanium oxide (TiO_x_) layer with nanohollows that shows a large increase in current of 142% under UV illumination provided by a 365 nm LED at a power of 83 mW. Based on AFM and SEM, the TiO_x_ grains formed in the step-bias anodic oxidation were found to lead to nanohollow generation. Results obtained from EDS mapping, HR-TEM and XPS all verified the TiO_x_ composition and supported nanohollow formation. The nanohollows formed in a thin TiO_x_ layer can lead to a high surface roughness and photon absorbance for photocurrent generation. With this step-bias anodic oxidation methodology, TiO_x_ with nanohollows can be obtained easily without any extra cost for realizing a high current under photoelectrochemical measurements that shows potential for electrochemical-based sensing applications.

## 1. Introduction

Photosensitive metal-oxide semiconductors with different energy band gaps (Eg) have been studied for many applications, including solar cells [1,2], photodetectors [3,4], photocatalysts [5,6], water splitting [7,8] and photoelectrochemistry (PEC) [9,10]. In general, extra carriers can be generated based on the absorption of photons with an energy higher than the band gap of a semiconductor (e.g., hν ≥ Eg) [11]. In electronic devices, such as solar cells and photodetectors, the photon-to-current efficiency and frequency response determined for photosensitive semiconductors can be considered the key performance parameters. In electrochemical devices, oxidation and reduction occur on the surfaces of photosensitive electrodes with the help of extra photoinduced charged carriers, which can be used to enhance chemical reactions and sensing performance. Therefore, the most common materials, including titanium oxide (TiO_2_) [12,13], ferric oxide (Fe_2_O_3_) [14,15] and zinc oxide (ZnO) [16,17], have been investigated widely for decades due to their natural abundance, high chemical stabilities, low costs and low toxicities, especially for realizing superior performance by means of various nanostructures [18]. TiO_2_ is the most promising material due to its various fabrication techniques and biocompatibility [19]. In general, self-organized nanotube arrays of these kinds of materials have the advantages of high surface areas fabricated by using the electrochemical anodization of a metal foil that can be obtained with specific bias-setting conditions and electrolytes [20,21,22]. For example, highly ordered TiO_2_ nanotube arrays were fabricated using titanium (Ti) foils at 35 V in a solution of 0.25 wt% of NH_4_F and 0.75 wt% of H_2_O in ethylene glycol [23]. In this way, TiO_2_ nanotubes with inner diameters and wall thicknesses of 130 and 15 nm, respectively, were created [24]. These kinds of electrodes are undesirable for applications since only a pure metal foil can be used as the substrate. However, transparent backside electrodes integrated with nanotube arrays are highly desirable in photovoltaics and photoelectrochemical-cell biorelevant applications due to short charge-transportation lengths and high light-harvesting efficiencies [25]. According to their effective and extra signals, photoelectrochemical biosensors with the advantages of high signal-to-noise ratios, good repeatabilities, low costs and simple instrumentations have attracted extensive interest in biology [26], medicine [27] and environmental monitoring [28]. In previous studies, TiO_2_ nanorods were prepared on FTO glass by using a hydrothermal method in a solution containing an equal ratio of 37 wt% of HCl and deionized (DI) water [29]. Titanium iso-propoxide was injected into the prepared solution, which we stored in a Teflon-coated container fixed with an autoclave at 150 °C for 6 h. This developed electrode was proven to be applicable to a photoelectrochemical sensor for beta-amyloid peptide detection. In addition, electrochemical anodization is an easy and efficient method for growing self-organized nanotubes that has been commonly investigated for decades. With the assistance of an electrical field and F^−^ ions in an electrolyte, Ti-based oxidation can be obtained with the structures of nanotubes or nanopores [30]. Recently, the anodization of Ti thin films deposited on glass substrates through a sputtering modification was studied for water splitting [25,31] and electrochromic devices [32]. Compared to a Ti layer deposited by an evaporator, TiO_2_ nanotubes formed on a sputtered Ti layer have five-fold higher photocurrent densities [25]. However, the nanotubes’ adhesion to glass substrates and pinholes during their growth is still a concern in real applications [25]. To avoid the cracking or peeling of TiO_2_ nanotubes, extra efforts for the modification process of Ti deposition [25,31] and post treatments, which limit their flexibility in real applications, have been addressed [33].

Based on previous literature [34], TiO_2_ nanotubes fabricated on a Ti foil by using electrochemical anodic oxidation can be used for photoelectrochemical measurements with signal enhancements [35]. However, these fabricated TiO_2_ nanotubes show no clear oxidation and reduction peaks in CV measurements due to their natural electrochemical properties [36] and potential biodamage due to front-side ultraviolet (UV) illumination during the photoelectrochemical operation [37]. For this work, a new process flow of electrode fabrication using a sputtered Ti thin film deposited on FTO glass and following different settings for anodic oxidation was designed to overcome the limitations of biosensing applications based on conventional Ti foils. The concept involved completely oxidizing the entire Ti layer into TiO_x_ for realizing a better photoelectrochemical response. Therefore, Ti films with different thicknesses and step biases for anodic oxidation were investigated for TiO_x_ formation with the creation of nanostructures, and their electrochemical and photoelectrochemical behaviors were characterized. Detailed material analyses were performed to obtain a clear understanding of the fabricated TiO_x_ layer with embedded nanohollows.

## 2. Materials and Methods

### 2.1. Electrode Fabrication

**Figure 1:**
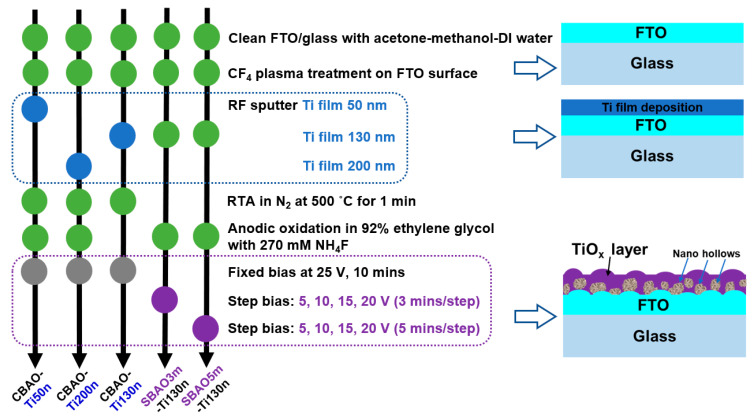
The detailed process flow for all experimental groups with the corresponding cross-sectional schematic plot.

To fabricate a reliable electrode with TiO_x_ nanostructures with the possibility of back-side illumination for reducing damage to biospecies, fluorine-doped tin oxide (FTO) glass substrates (NSG TEC A7, Pilkington, Lathom, UK) with sheet resistances of 10 Ω/sq were selected and cut to dimensions of 25 mm × 10 mm × 1.1 mm for sputtered-titanium (Ti) layer depositions and subsequent electrochemical anodic oxidations. FTO glass substrates were first cleaned in solvents in the sequence of acetone, methanol and DI water for 10 min per solution with an ultrasonication process. Then, FTO glass substrates were dried with a nitrogen (N_2_) stream. To obtain better adhesion between the Ti film and FTO glass substrates, the FTO glass substrates were pretreated with CF_4_ plasma before titanium layer deposition. The CF_4_ plasma treatment was performed using inductively coupled plasma-reactive ion etching (ICP-RIE; KD-ICP/RIE, Kao Duen, New Taipei City, Taiwan) with generation-power, bias-power, period, gas flow-rate and pressure settings of 300 W, 50 W, 3 min, 20 sccm and 100 mTorr, respectively. To reduce ion bombardment at the surface, a stainless shielding filter was placed on top of the FTO glass substrate [38]. The above cleaning and CF_4_ plasma procedure not only removed surface contamination but also created a good surface quality for subsequent Ti thin film depositions. By using a radio frequency (RF) magnetron sputtering system with a titanium target with a purity of 99.99% (TIC36KRD, Summit, Taipei, Taiwan), Ti thin films with different thicknesses were deposited onto an FTO glass substrate controlled by a time mode according to a deposition rate calculated from previous test experiments and checked by using a surface profiler (Alpha Step DeltakXT, Bruker, Billerica, MA, USA). Before the deposition process, the chamber was pumped down to a base pressure of 10^−6^ mTorr using a turbo-molecular pump. The flow rate of argon (Ar), RF power and pressure for Ti deposition were 40 sccm, 70 W and 8 mTorr, respectively. The substrate temperature was increased to 300 °C to improve the quality of the Ti film deposition. After sputtering Ti films on FTO glass, the Ti thin films were densified by using rapid thermal annealing (RTA; RTA1000M-V, SJ High Tech Co., Taipei, Taiwan) under ambient N_2_ at 500 °C for 1 min to improve the film quality. Figure 1 shows the step-by-step process flow for all experimental groups, including Ti thickness and anodic oxidation, for TiO_x_ electrode fabrication.

**Figure 2:**
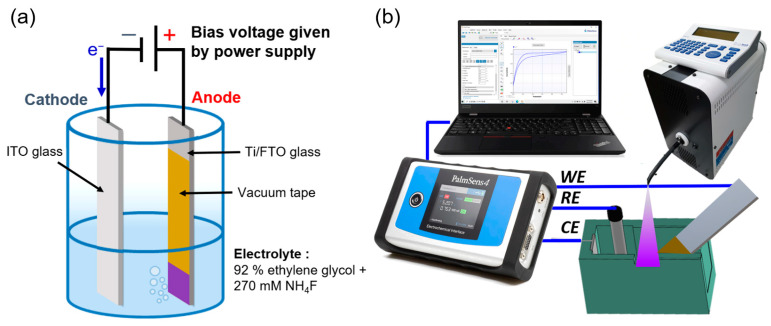
(**a**) The experimental setting for anodic oxidation, including electrolyte, counter and working electrodes, and (**b**) the photoelectrochemical measurement system setup.

The 2-electrode electrochemical setup was used to anodize the Ti layer deposited onto the FTO glass, as shown in Figure 2a. All the anodization experiments were performed at room temperature in a conventional two-electrode system using a Ti/FTO glass substrate as the working electrode and ITO glass (RLO-I7, Ruilong, Miaoli, Taiwan) with dimensions of 6 cm × 2.6 cm × 0.7 mm and a resistivity of 5 Ω/sq as the counter electrode. Based on our preliminary data, the results obtained for the ITO counter electrodes are similar to those obtained for platinum (Pt) electrodes. Therefore, disposable ITO glass was used to replace the conventional Pt electrode to obtain the same fresh surface for the counter electrode in the anodic oxidation process. The Ti film was anodized in a mixed solution of 92% ethylene glycol and 270 mM ammonium fluoride (NH_4_F) [39]. During the electrochemical anodizing process, a controllable DC power source was used to supply the required constant bias voltage or step-bias voltage. To study the efficiency of anodization, various Ti film thicknesses, including 50 nm, 130 nm and 200 nm, were studied first in constant-bias anodic-oxidation (CBAO) experiments with a fixed bias of 25 V for 10 min. These 3 groups were named CBAO-Ti50n, CBAO-Ti130n and CBAO-Ti200n, respectively. Then, in the second part of the anodic-oxidation bias voltage evaluation, all experimental groups with fixed Ti thicknesses of 130 nm were treated with a step-bias anodic oxidation (SBAO) method using the following voltage settings: increasing the step voltage of 5 V, 10 V, 15 V and 20 V and a period of 3 or 5 min for each step of voltage. These 2 experimental groups were named SBAO3m-Ti130n and SBAO5m-Ti130n, respectively.

### 2.2. Photoelectrochemical Response

A standard three-electrode system with a commercial potentiostat (PalmSens4, PalmSens, Houten, The Netherlands) was established for photoelectrochemical measurements, as shown in Figure 2b. The three electrodes that were used were as follows: a nanostructured TiO_x_-layer grown on Ti/FTO glass encapsulated with vacuum tape for an area of 1.0 × 1.0 cm^2^ as the working electrode, a commercial Ag/AgCl reference electrode (ALS, RE-1S, Tokyo, Japan) and a platinum (Pt) sheet (Pt10101, ING-JING, New Taipei City, Taiwan) with dimensions of 1.0 × 1.0 cm^2^ as the counter electrode. A cyclic voltammetry (CV) measurement was performed in a 0.1X PBS background solution containing 5 mM of ferricyanide (K_3_[Fe(CN)_6_]). The scan rate and range of CV measurement were 0.1 V/s and from −0.6 V to 0.8 V, respectively. To generate an extra photocurrent for the CV measurement, ultraviolet (UV) illumination at a wavelength of 365 nm with a power of 83 mW was applied to the fabricated TiO_x_ nanostructured electrode using an LED (pE-4000, CoolLED, Andover, UK). The photoelectrochemical performance of the prepared electrodes was further tested by conducting linear-sweep voltammetry (LSV) measurements under chopped UV illumination with a power of 83 mW and an on/off cycle with a period of 56 s in a 0.1X PBS solution. A scan rate of 0.05 V/s, with a scan range of −0.4 V to 1.0 V, was used to compare measurements with or without continuous UV illumination.

### 2.3. Material Characterization

To obtain a detailed understanding of the correlation between the material properties and electrochemical behavior of this novel TiO_x_ nanostructure layer, various material analyses were performed for samples using the same process conditions. First, the morphologies and microstructures of the fabricated samples were characterized by using an atomic-force microscope (AFM; Nanoview 1000, Utek Material, Taipei, Taiwan). During each measurement, the distance between the tip and the sample was controlled in tapping mode to scan the surfaces for all samples. AFM images and calculated average surface-roughness (Ra) values were obtained using the data-analysis software WSxM (Version 5 develop 8.4, Nanotec Electronica, Madrid, Spain). Additionally, scanning electron microscopy (SEM) was performed to compare AFM results obtained through investigation on a top view of TiO_x_/FTO glass layers. Furthermore, to investigate the cross-sectional layer distribution and nanostructure, a dual-focused ion beam (FIB; Versa 3D, FEI Company, Hillsboro, OR, USA) was used to slice the whole structure first, and then, a field-emission scanning-electron microscope (FE-SEM; SU8220, Hitachi, Tokyo, Japan) was used to obtain images of the nanostructures. The lattice structure and elemental mapping analysis were observed using high-resolution transmission electron microscopy (HR-TEM; JEM-2100, JEOL, Tokyo, Japan) operated at 200 kV with a point resolution of 0.19 nm.

X-ray diffraction analysis (XRD; PANalytical Empyrean, Malvern Panalytical, Almelo, The Netherlands) with Cu Kα radiation (λ = 0.15406 nm) was performed to observe the crystalline phase with parameters of 45 kV, 40 mA and a 2θ range of 10°–70°. The compositions and chemistries of the nanostructured TiO_x_ layers were investigated by using X-ray photoelectron spectroscopy (XPS; PHI 5000 VersaProbe III, ULVAC-PHI Inc., Chigasaki, Japan) performed in an ultra-high vacuum chamber with an Al Kα (hν = 1486.6 eV) monochromatic X-ray source. The binding energies were calibrated with reference to the C *1s* peak at 284.8 eV. A detailed analysis and comparison are presented in the results and discussion section.

## 3. Results

### 3.1. Material Characterization

**Figure 3:**
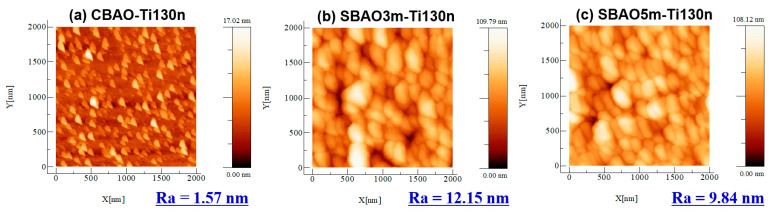
AFM image of the surface morphology for the sample with a 130 nm-thick Ti/FTO glass with different anodization conditions: (**a**) CBAO-Ti130n, (**b**) SBAO3m-Ti130n and (**c**) SBAO5m-Ti130n.

**Figure 4:**
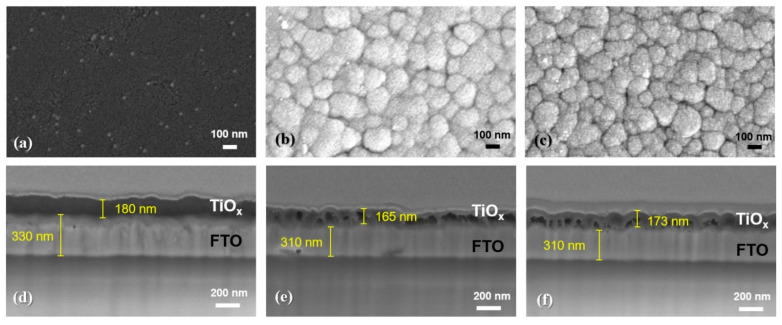
Top-view SEM images with the same magnitudes and scale bars of 100 nm for the surfaces of samples prepared with 130 nm-thick Ti/FTO glass with different anodization conditions: (**a**) CBAO-Ti130n, (**b**) SBAO3m-Ti130n and (**c**) SBAO5m-Ti130n. Cross-sectional SEM image with the same magnitude and scale bar of 200 nm for (**d**) CBAO-Ti130n, (**e**) SBAO3m-Ti130n and (**f**) SBAO5m-Ti130n samples.

**Figure 5:**
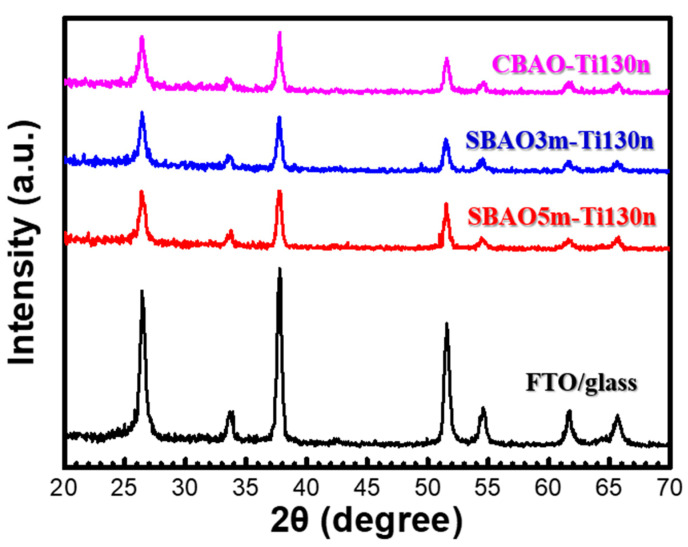
XRD spectra obtained for the CBAO-Ti130n, SBAO3m-Ti130n, SBAO5m-Ti130n and FTO/glass samples.

To obtain a clear understanding of the effects of anodic oxidation on Ti/FTO glass samples, various material analyses, including AFM, SEM, TEM, EDS and XPS, were performed. After an anodic oxidation, all the samples were subjected to AFM measurements to confirm their surface morphologies and surface roughnesses. AFM images of CBAO-Ti130n, SBAO3m-Ti130n and SBAO5m-Ti130n samples are shown in Figure 3a–c, respectively. For the CBAO-Ti130n sample, some small roughnesses were observed with an average roughness (Ra) of 1.57 nm. With step-bias anodic oxidation, the surface roughnesses of the SBAO3m-Ti130n and SBAO5m-Ti130n samples increased significantly to 12.15 nm and 9.84 nm, respectively. Moreover, some nanometer-scale grains were observed in both the samples. To obtain a better understanding, top-view and cross-sectional-view SEM images were captured for all the three samples. The top-view images of CBAO-Ti130n, SBAO3m-Ti130n and SBAO5m-Ti130n are shown in Figure 4a–c, respectively. In general, the top-view SEM images well matched the AFM results obtained for the surface roughnesses and morphologies. The CBAO-Ti130n sample showed a flat surface but with some small particles. With step-bias anodic oxidation, grains with clear boundaries formed on both the SBAO3m-Ti130n and SBAO5m-Ti130n samples. The grains and their boundaries in SBAO samples result in a high surface roughness, which matched the increase in Ra measured by AFM. To confirm the potential mechanism of grain formation through anodic oxidation, cross-sectional SEM images of CBAO-Ti130n, SBAO3m-Ti130n and SBAO5m-Ti130n are shown in Figure 4d–f, respectively. For the CBAO-Ti130n sample, it can be observed that the TiO_x_, the FTO and the glass layers stacked from top to bottom all met the thickness and distribution requirements of the process conditions. For the SBAO groups, an FTO layer with a thickness of approximately 310 nm and a TiO_x_ layer with obvious nanohollows can be observed in Figure 4e,f. The total thicknesses of the TiO_x_ layer for the SBAO3m-Ti130n and SBAO5m-Ti130n samples were approximately 165 nm and 173 nm, respectively. This can be used to support the small difference in the level of the anodic oxidation of the Ti layer that was 130 nm over different time periods from 3 min to 5 min. These nanohollows with dimensions of approximately 100 nm in height can lead to grain formation and an increased surface roughness. To confirm the correlation between roughness and crystallization, the XRD spectra for CBAO-Ti130n, SBAO3m-Ti130n, SBAO5m-Ti130n and FTO/glass (e.g., the control sample of substrate) are presented in Figure 5. No clear crystallization peak of titanium oxide was found for any of the three samples, but all the peaks can be referred to the FTO [40]. It can be concluded that the crystallization of TiO_x_ is not strong and shows a more amorphous structure [41]. It can be reasonably assumed that the high surface roughness in the SBAO5m-Ti130n group is mainly due to the nanohollow structure and is not due to the orientation of the crystallization.

**Figure 6:**
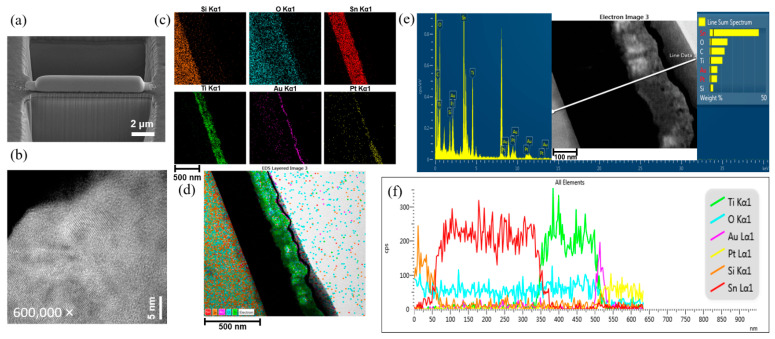
(**a**) SEM image recorded after FIB cutting, (**b**) HR-TEM image with a scale bar of 5 nm, (**c**) separated EDS mapping of different atoms, including Si, Sn, O, Ti, Au and Pt, with a scale bar of 500 nm, (**d**) stacked EDS mapping for all five atoms, including Sn, Si, Au, O and Ti, with a scale bar of 500 nm, (**e**) EDS spectrum obtained via line scanning, shown as a marked line in the inset and (**f**) intensity distribution of the scanning line obtained for all six atoms mentioned above for the SBAO3m-Ti130n sample with embedded nanohollows in the anodic oxidized TiO_x_ layer.

**Figure 7:**
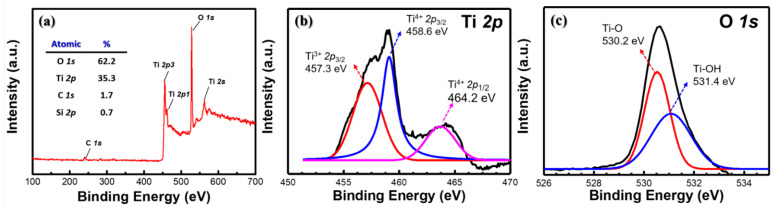
(**a**) Full-scale, (**b**) high-resolution Ti *2p* and (**c**) O *1s* XPS spectra obtained for the SBAO3m-Ti130n sample with embedded nanohollows in the anodic oxidized TiO_x_ layer.

To further confirm the composition of the layer distribution, an FIB was used to cut the SBAO3m-Ti130n sample into a thin slice for HR-TEM imaging with a corresponding EDS mapping. The title-view SEM image was captured after the FIB cutting, as shown in Figure 6a. An e-beam-deposited Pt line was used to shield the area, and then, the surrounding area was cut by using a focused ion beam. This sliced sample was used for a TEM analysis. As shown in Figure 6b, a periodic arrangement of the Ti layer with different orientations was clearly observed under a magnification of 600 K. To evaluate the composition of this sliced sample, the separated elemental mapping images recorded by using EDS for different atoms, including Si, O, Sn, Ti, Au and Pt, are shown in Figure 6c. The area of Si atoms with respect to the glass can be observed from the left side. Then, the area of Sn and Ti atoms can be seen from the FTO and TiO_x_. In these three areas, O atoms can also be found. The area of each kind of atom can be clearly distinguished. On the right side, an area of thin Au and Pt layers can be observed, which originated from the post-deposited conductive metal layer and shielding layer used for FIB, respectively. Then, a stacked mapping image of all six atoms was rearranged, as shown in Figure 6d. The area of the TiO_x_ layer can be clearly observed and matched to the previous SEM image, as shown in Figure 4e. Some small white areas can be found in the TiO_x_ layer, as shown in Figure 6d, which can be attributed to the nanohollows. To further check the atom distribution, the line scanning for an elemental analysis using EDS is also presented for all six atoms, as shown in Figure 6e. The EDS scanning line is marked in the TEM image, as shown in the inset of Figure 6e. The atom distribution across the scanning line for all six atoms is shown in Figure 6f. From approximately 0 nm to 50 nm, a high intensity of Si and O atoms in the glass substrate (e.g., SiO_2_) could be expected. An increase in the intensity of the Sn atoms accompanied by a decrease in the intensity of the Si atoms from 50 nm to 80 nm could be attributed to the interface between the FTO and the glass layer. The total thickness of the FTO can be estimated to be the depth with a high intensity of Sn from 50 nm to 350 nm. Then, the Ti concentration increased for the TiO_x_ layer from 350 nm to 500 nm. A Au layer could be found on top of the TiO_x_ layer. Finally, a Pt shielding layer prepared in the FIB process was observed. Based on these EDS elemental-mapping and line-scanning results, the thickness of each layer was found to be approximately consistent with the SEM images and fabrication processes. In the meantime, some peaks in the green line due to a high concentration of Ti atoms in the depth analysis and some white areas in the stacked mapping image were observed, which provided strong evidence for TiO_x_ formation and nanohollows, respectively. All the results obtained from the SEM, TEM and EDS mapping were fully matched to the expected results for the SBAO3m-Ti130n sample. It can be inferred with high confidence that TiO_x_ nanohollows were formed during the step-bias anodic oxidation of the Ti layers in the developed experiments. To confirm the composition of this fabricated nano-hollowed TiO_x_ layer, XPS was used to study the chemical states of the surface of this sample for all the possible atoms. As shown in Figure 7a, strong Ti and O peaks can be found from the surface XPS analysis. The Ti and O atomic ratios were 35.3% and 62.2%, respectively. To analyze the chemical binding of this TiO_x_ layer in detail, the XPS spectra for Ti *2p* and O *1s*, measured for the SBAO3m-Ti130n sample, were rearranged with de-convolutions, as shown in Figure 7b,c, respectively. The Ti *2p*_3/2_ and Ti *2p*_1/2_ binding peaks were located at 458.6 eV and 464.2 eV, respectively. This result matches published results, which demonstrates the presence of Ti^4+^ in the TiO_x_ lattice [42]. A Ti *2p* peak appeared at 457.3 eV, which can be attributed to the small amount of Ti^3+^ present in the TiO_x_ layer [42]. The O *1s* spectrum could be fitted with two peaks, as shown in Figure 7c. The peak at 530.2 eV can be attributed to Ti-O bonds in the TiO_x_ lattice, and the peak at 531.4 eV can be attributed to surface hydroxyl groups or adsorbed oxygen [43].

### 3.2. Photoelectrochemical Measurements

#### 3.2.1. Effect of Ti Thickness

**Figure 8:**
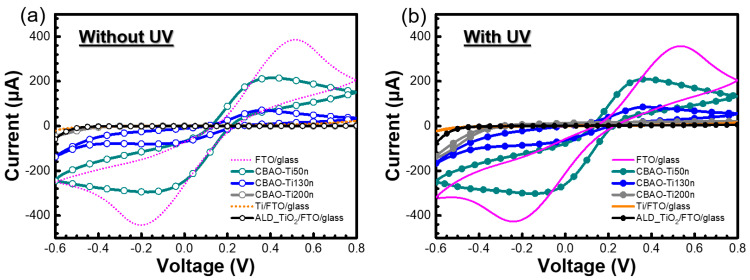
CV curves measured for different electrodes, including FTO glass, CBAO-Ti50n, CBAO-Ti130n, CBAO-Ti200n, Ti/FTO glass and ALD TiO_2_/FTO glass in 0.1X PBS solution containing 5 mM ferricyanide K_3_[Fe(CN)_6_] at a scan rate of 0.1 V/s (**a**) without and (**b**) with UV illumination at wavelength of 365 nm and power of 83 mW.

To evaluate the electrochemical and photoelectrochemical behaviors, cyclic voltammetry (CV) curves were collected for the experimental groups with different Ti thicknesses with a constant bias anodic oxidation at 25 V for 10 min. As shown in Figure 8a,b, CV curves with and without the UV illumination of CBAO-Ti50n, CBAO-Ti130n and CBAO-Ti200n could be clearly observed. To evaluate the correlation between the electrode status and CV behavior, CV curves for FTO glass, Ti/FTO glass and TiO_2_ deposited through atomic-layer deposition (ALD) on an FTO glass electrode were also presented as reference groups. First, CV curves without UV illumination presented the typical behaviors, which could be used to check the surface material and its oxidation and reduction peaks, as shown in Figure 8a. The sample with the FTO surface had a typical CV curve and clear peaks, and the samples with the Ti or ALD TiO_2_ surfaces both showed no oxidation nor reduction peaks, which matched the results reported in the literature [44,45]. For the CBAO-Ti50n sample, the peak currents for oxidation and reduction both showed similar behaviors but with smaller values than those obtained for the FTO sample. In this sample, the electrochemical behavior was found to be close to that of FTO, which suggests that some parts of FTO under the Ti layer were exposed after CBAO. This also means that the Ti layer can be partly peeled off from the FTO layer during CBAO. A Ti thickness of 50 nm may not be sufficient for a CBAO treatment at 25 V for 10 min. For samples with thicker Ti layers, the current, typical oxidation and reduction peaks were all reduced with the same anodic oxidation on Ti surfaces. In the CBAO-Ti130n group, the peak currents were reduced to approximately 59% compared to those obtained for CBAO-Ti50n. For the CBAO-Ti200n group, no peak could be found, which can be attributed to the surface composed of more TiO_x_ that resulted in a lower conductivity for a smaller current. To confirm the photoelectrochemical behaviors of the same groups, CV measurements with UV illumination at a wavelength of 365 nm with a power of 83 mW were performed, and the results are shown in Figure 8b. Due to the energy gap and absorbance of TiO_x_, UV illumination at 365 nm can be absorbed by TiO_x_ and then transferred to an extra photocurrent [46]. However, a photocurrent will not be induced in FTO or Ti samples under UV illumination due to a lack of absorbance and the energy band gaps of these materials [47]. No increment in current was found for the CBAO-Ti50n sample with UV illumination, which can be attributed to a very rare TiO_x_ layer that was formed, with the sample’s response mainly dominated by FTO. This behavior was consistent with that expected from the electrochemical characterization shown in Figure 8a. As the Ti thickness increased, the current under illumination was higher than the current without illumination, which is shown for the sample with Ti thicknesses of 130 nm and 200 nm. The maximum current in the CBAO-Ti200n sample was not high enough under UV illumination. To improve the photo responses of the CV curves, a Ti thickness of 130 nm was selected as a first fix in the process condition. Then, the anodic oxidation procedure was modified as shown in the second part of the figure to reduce the possibility of Ti peeling off from the FTO surface and to ensure that more Ti remained on the FTO to be oxidized as a TiO_x_ layer.

#### 3.2.2. Effects of Anodic Oxidation Conditions

**Figure 9:**
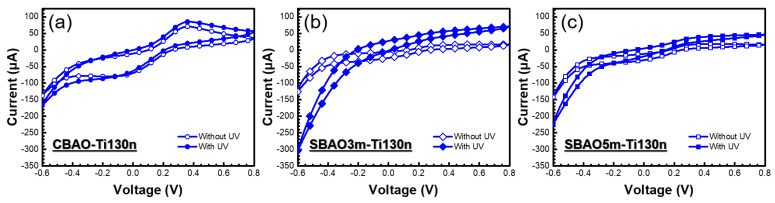
CV curves measured with and without UV illumination for different electrodes, including (**a**) CBAO-Ti130n, (**b**) SBAO3m-Ti130n and (**c**) SBAO5m-Ti130n electrodes, in 0.1X PBS solution containing 5 mM K_3_[Fe(CN)_6_] at a scan rate of 0.1 V/s.

**Figure 10:**
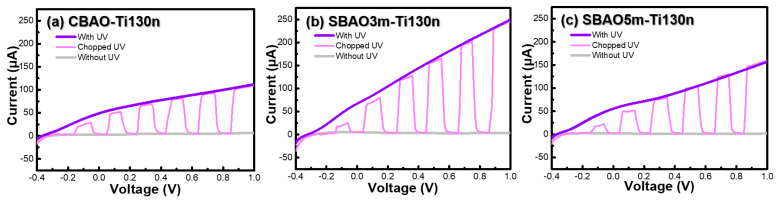
Different LSV curves measured with, without, and with chopped UV illumination at 365 nm with a power of 83 mW in 0.1X PBS solution at a scan rate of 0.05 V/s from −0.4 V to 1 V for (**a**) CBAO-Ti130n, (**b**) SBAO3m-Ti130n and (**c**) SBAO5m-Ti130n samples, respectively.

Due to the partial peeling off of the 50-nm-thick Ti layer under constant bias anodic oxidation and the sample with a 200-nm-thick Ti layer having a very low current, a modification in the anodic oxidation with gradual step increases of bias voltage, called step-bias anodic oxidation, was applied to samples with Ti thicknesses of 130 nm. As shown in Figure 9a–c, CV curves with and without UV illumination for CBAO-Ti130n, SBAO3m-Ti130n and SBAO5m-Ti130n, respectively, can be clearly observed. With the SBAO procedure, the current became higher than that for CBAO, both shown at 3 min and 5 min, which can be attributed to an increased oxidation of the Ti layer. It can be clearly observed that the SBAO groups both showed higher current increments with UV illumination, which can be attributed to the effects of TiO_x_ formation. Moreover, the oxidation and reduction peaks could not be found in SBAO groups due to the TiO_x_’s material behavior, which was similar to the results obtained from Section 3.2.1. The current ratios at −0.6 V for SBAO3m-Ti130n and SBAO5m-Ti130n increased by 142% and 56%, respectively. It can be concluded that the photon-to-current efficiency increased for the sample prepared by SBAO. The highest photocurrent could be found in the SBAO3m-Ti130n group. To further evaluate the photochemical behavior, linear-sweeping voltammetry (LSV) curves were obtained for these three samples to study the separation of photo-generated electron-hole pairs based on the increased photocurrent. LSV was performed at a scan rate of 0.05 V/s and then with different conditions, including chopped and with and without UV illumination at 365 nm with a power of 83 mW. As shown in Figure 10a, the current measured without the UV illumination was very low, and the current measured with the UV illumination increased with an increasing bias voltage. For chopped illumination with a period of 56 sec at a duty cycle of 50%, the on-off behavior of the photocurrent could be clearly observed between the curve with and without UV illumination. The curves obtained for the same measurement conditions for the SBAO3m-Ti130n and SBAO5m-Ti130n samples are shown in Figure 10b,c, respectively. The highest photocurrent and the fastest transient response could be observed in the SBAO5m-Ti130n sample. The response and recovery times of the photoresponse were within a few ms, which matches the published results for TiO_x_ [48,49,50]. With this great enhancement in current shown in both the CV and LSV measurements, TiO_x_ prepared by using SBAO can be considered a potential candidate for photocatalyst and photoelectrochemical applications. Based on all the material analyses, step-bias anodic oxidation on a Ti layer was proven to create a TiO_x_ layer composited with nanohollows following an increase in surface roughness and a 142% increment in the photocurrent under UV illumination at 365 nm with a power of 83 mW. This proposed process of step-bias anodic oxidation with only simple modifications to the parameter settings not only improves the stability for conventional fixed-bias anodic oxidation but also leads to a superior photoresponse, which can be suggested for applications in photocatalysis and photoelectrochemistry. In particular, photoelectrochemical sensing could be enhanced through back-side illumination through a transparent substrate composed of FTO glass to reduce photo-induced damage for biospecies detection, such as antibody and cell detection.

## 4. Conclusions

In summary, a TiO_x_ layer with nanohollow structures was successfully grown onto Ti/FTO glass by using the variable step-bias anodic oxidation of a Ti layer. To obtain a detailed insight into the physicochemical material properties and electrochemical features of the novel TiO_x_ nanostructure layer, various material analyses were performed for samples using the same process conditions, including AFM, SEM, XRD, HR-TEM, EDS and XPS. After an optimization of the experiments, a Ti layer thickness of 130 nm and step-bias settings of 5 V, 10 V, 15 V and 20 V for 3 min per step are suggested, which can result in a stable TiO_x_ layer with a thickness of approximately 160 nm. The average surface roughness of this layer is 12.15 nm. Nanohollows with diameters of approximately 100 nm were observed by using SEM. The current ratio was increased by 142% in photoelectrochemical measurements of this fabricated TiO_x_ layer embedded with nanohollows under UV illumination at a power of 83 mW. Further applications of this developed nanohollow-structured TiO_x_ electrode are suggested for photoelectrochemical biosensing and photocatalysis.

## Data Availability

The data presented in this study are available upon request from the corresponding authors.
